# SENSORY LOSS IN AGING: SPOTLIGHTING JUNIOR AND NEW GSA INVESTIGATORS IN THE SENSORY HEALTH SPECIAL INTEREST GROUP

**DOI:** 10.1093/geroni/igad104.0628

**Published:** 2023-12-21

**Authors:** Jennifer Deal, Heather Whitson

## Abstract

Our senses (hearing, vision, touch, taste, smell) are the means by which our brains perceive our environment, allowing us to engage and connect with our world. Recommendations for maintaining brain health and function with age include maintaining strong social connections and participating in intellectually stimulating mental and physical activities. However, our senses are what allow us to engage in these activities. Sensory loss is common in older adults, with over 2/3 of adults over age 55 experiencing loss in multiple senses. The goal of the Sensory Health Special Interest Group (SIG) is to foster collaboration and dissemination of research and clinical/educational resources for professionals interested in sensory health and the effects of sensory impairments on the overall health and well-being of older adults. This session will meet this goal by highlighting work that demonstrates the importance of sensory health with age conducted by outstanding Sensory Health SIG investigators who are early in their career path or who are new to GSA. We will start by discussing the importance of olfaction for mental health over time in older adults and vision for markers of neurodegeneration. We will then have three presentations that consider multiple risk factors, including sensory loss, for cognitive function. First, we will present on the moderation of the association between hearing loss and dementia by sleep. We will then present on the association between multisensory loss and cognitive test performance, and finally, how the inclusion of sensorimotor measures can improve prediction of subsequent neurodegeneration and cognitive impairment. This is a Sensory Health Interest Group Sponsored Symposium.

